# ‘I think I will need help’: A systematic review of who facilitates the recovery from gender‐based violence and how they do so

**DOI:** 10.1111/hex.13157

**Published:** 2020-11-20

**Authors:** Patricia Melgar Alcantud, Roger Campdepadrós‐Cullell, Concepció Fuentes‐Pumarola, Elena Mut‐Montalvà

**Affiliations:** ^1^ Social Work and Social Services Unit Department of Pedagogy University of Girona Girona Spain; ^2^ Sociology Unit Department of Business Studies University of Girona Girona Spain; ^3^ Nursing Unit Department of Nursing University of Girona Girona Spain; ^4^ Social Work and Social Services Unit Department of Social Work and Social Services Valencia Spain

**Keywords:** gender‐based violence, recovery, solidarity networks, support

## Abstract

**Context:**

A key to advancing the eradication of gender‐based violence (GBV) is knowing how to recover from it.

**Objective:**

To identify the changes that are indicators of having overcome GBV and determine the role of various support networks.

**Search strategy:**

We systematically searched Web of Science.

**Inclusion criteria:**

Publications whose abstracts contained a minimum of two of the following words: (a) support or network or solidarity, (b) violence and (c) recovery or healing.

**Data extraction and synthesis:**

Of the 273 documents retrieved, 52 were used using a narrative synthesis approach.

**Main results:**

For recovery, women must reconnect with themselves, with their environment and with the world in general. Doing so requires support from both formal and informal networks. The intervention of other people is a common element in successful recovery processes. We identify three requirements for the intervention of these support networks to be effective: not blaming the victim, making women part of their own recovery process by showing them their own transformation potential and promoting reflection on the socialization they have experienced in their affective‐sexual relationships.

**Conclusions:**

There are multiple benefits to having formal or informal support during recovery. In informal networks, raising awareness and providing training are insufficient for promoting active support. Instead, the Second Order of Sexual Harassment must specifically be combatted.

**Public contribution:**

From the authors' previous research, harassed women and survivors have underlined the necessity to identify indicators of recovery and which kind of support has an impact on it.

## 1 INTRODUCTION

This article is framed into the research *SOL NET Solidarity networks with impact on gender‐based violence victims’ recovery processes*.^1^ Previous research has identified that knowing how to quit and recover from gender‐based violence (GBV) is a key element to advance on its eradication.^2^, ^3^ Likewise, recent advances on GBV prevention have brought to the fore the need of promoting the community's active role in preventing this violence from happen or to palliate its effects once it has already taken place.^4^, ^5^ Thus, the main objective of SOL.NET research is to identify the common elements of the solidarity actions that contribute to the recovery process of women who have experienced GBV. The present article provides a theoretical framework regarding the transformations observed in the GBV survivors's lives as they progress through their recovery process. We also identify the role of support networks, especially informal networks, in this process. Thereby, based on the current state of the art, we contribute with those elements that show the social impact of the intervention on GBV survivors’ recovery. This will allow developing evidence‐based policies and guiding interventions that achieve social impact. An aspect that have been lately prioritized in Europe, specifically in Social Sciences.^6^

## 2 METHODOLOGY

In the preparation of this article, 263 publications indexed in the Web of Science database were initially selected. The search was narrowed to three topics: (a) support or network or solidarity, (b) violence, and (c) recovery or healing. No temporal filter or geographical localization was established. The 263 initially selected articles met the requirement of containing a minimum of two of the three topics in their abstracts. The abstracts of the 263 articles were read, and 71 articles were selected and analysed to determine the presence of a direct link with the topic of study: recovery from GBV. These articles were reviewed by at least two researchers, who selected those researches that both considered were appropriate to construct the theoretical framework. In some few particular cases, a third researcher view was needed to make the selection. Finally, the theoretical body presented here is based on the contributions of 52 documents. Our analysis also included documents that were repeatedly cited in the selected articles. Studies were included in the analysis when they were related directly to our focus or when the understanding or correct analysis of the initially selected articles required a direct reading of the theoretical frameworks they cited.

Is worth noting that most of the reviewed researches have been conducted in Western countries with Caucasian population, while three of them are from Democratic Republic of the Congo, New Zealand and Uganda.

The results of this review article are organized as it follows. First, there is a general explanation about GBV recovery, highlighting those elements that indicate a progress on the field; second, there is an analysis of the recuperation's first steps, that is, how the women under GBV carry out and develop the search for help; third, in this sense, there is a contribution of knowledge about the features of those supports that has social impact on the aforementioned recovery; and fourth, based on all of this evidence, we contribute with a set of orientations to guide interventions with women who are suffer GBV.

## 3 RECOVERY

Recovering from gender violence is a non‐linear process, and the women it affects can have very diverse characteristics. The consequences of having experienced violence can persist long after the relationship ends and vary widely, including mental health disorders, depression and substance abuse, among others.^7^ Specifically, the consequences include changes in how the women perceive themselves; for example, they may feel less competent than previously and inferior, with little control over their emotions. There are also feelings of shame, guilt and fear of being judged by others.^8^, ^9^, ^10^ This external judgement can pressure women to respond to society's expectations, to move away from being stigmatized as victims, and to stop appearing weak to people around them.^11^, ^12^, ^13^, ^14^, ^15^ These latter factors related to the woman's image play an important role in the recovery process since they shape her thoughts and feelings and influence her behaviour. Therefore, the ways in which women perceive social expectations and their image in the eyes of others will affect their help‐seeking behaviour and the outcome obtained and can even indicate the likelihood of revictimization or successful integration.^13^, ^14^

We identify two major challenges in the work of recovery: managing post‐traumatic symptoms^16^ and coping with the demands of everyday life, such as housing, food, clothing and medical needs.^17^, ^18^

Regarding the management of post‐traumatic symptoms, most of the related literature refers to the process of recovering from trauma in general. Fewer studies focus on analysing recovery after GBV. Regarding trauma in general, Judith Herman^19^ defines recovery as the act of rebuilding the self through reconnecting to oneself and reintegrating into one's environment. In the specific case of GBV, Landenburger^20^ defines recovery as reaching a new balance and finding new meaning in life once the violence has ended.

Referencing Hernan's definition,^19^ Sinko and Saint Arnault^21^ establish three major indicators of recovery from gender violence: reconnection with the self, with others and with the world. Reconnection with the self entails reclaiming one's identity (recovering self‐esteem, strength, overcoming doubts, etc), managing emotions, controlling physical symptoms and memories related to situations of violence, recovering control over one's life, and making decisions autonomously. Reconnection with others involves building and maintaining relationships, feeling a sense of belonging, perceiving a supportive environment, and becoming involved in the community. Reconnecting with the world consists of releasing negativity by constructing a positive view of the world and living a purposeful life by finding fulfilment and personal growth.

The contributions of Sinko, Burns, O'Halloran, and Saint Arnault^22^ and other authors^12^, ^23^, ^24^ reveal an important differentiation between surviving and creating a new life. Creating a new life is a step beyond surviving violence. It involves taking time to reflect and create a new identity distanced from violence. It is not only about surviving violence; it also involves important changes in how the individual views relationships, herself and her philosophy of life.^3^, ^23^, ^24^

Therefore, while not all women have the same opportunity to make structural changes to their lifestyles, the aforementioned research goes beyond structural changes to propose a process of reflection on the meaning the individual gives to her experiences, which entails a change in the meaning given to life and, especially, to feelings.^3^, ^22^, ^25^, ^26^, ^27^, ^28^ Specifically, to promote this self‐awareness and creation of meaning, Koegler et al^27^ propose empowerment and narrative self‐revelation.

Other strategies used to cope with recovery, in general, are exercising; escaping into nature; accessing mental health resources; overcoming self‐isolation to interact with others, especially other women who have survived gender violence; joining leisure, educational, or religious groups; volunteering within the community; focusing on one's children; and working to help others.^2^, ^21^

On the other hand, coping strategies can sometimes be maladaptive, creating unhealthy habits, such as addictions, that are difficult to break and that therefore hinder progress in recovery.

Successful recovery depends on diverse variables, although sinko and saint arnault^21^ highlight that the intentionality of women when taking action to recover is an essential element. Our analysis of different investigations emphasizes a second common element: the intervention of other people. We have not found any research that confirms that successful recovery can be achieved alone. In contrast, studies agree on the need to break free from isolation and find support for meeting one's needs, sharing experiences, and dismantling negative self‐perceptions.^13^, ^21^, ^25^

## 4 SEEKING HELP: FIRST INTERACTIONS

Women do not usually keep the violence they experience hidden. In most cases, they look for someone to confide in. In the research carried out by Fanslow and Robinson,^29^ 75% of women told someone about the violence they were experiencing, and they usually turned to informal sources of support, such as family and friends. Ansara and Hindin^30^ found similar results. In the latter case, 87% of women reported confiding in at least one informal source. The percentages were similar in a European survey carried out in 28 countries with 42 000 women. In cases of physical or sexual violence from a partner or ex‐partner, 67% of women went to the police or other services or told someone about it.^31^ These findings leave 30% of women who do not report it, a proportion that must not be ignored. In an analysis of gender violence in a university context, Valls, Puigvert, Melgar and Garcia‐Yeste^32^ concluded that an adverse environment that blames women and does not support them discourages them from reporting gender violence or seeking help.

We find an extensive scientific literature that has analysed the decision pathways that women in a situation of gender violence follow, especially when they are trying to end the relationship. Liang, Goodman, Tummala‐Narra and Weintraub^33^ outline three stages of decision making: defining the problem, deciding to seek help and selecting a source of support. During these stages, as we indicated in the previous section, the response women receive when seeking help plays a critical role. These responses can help women define the problem, move forward in the attempt to end the relationship, and help in the recovery progress, or they can have the opposite effect. For example, women who receive supportive responses from the first people they confide in about the violence they are experiencing show greater confidence and are more likely to seek help in the future.^34^ In contrast, fear that a confidante will insist that the woman end the relationship or will impose their own judgement about what the victim is experiencing is an important barrier in help‐seeking processes.^8^ In such situations, some of the patterns the woman has already experienced during the violent relationship repeat themselves, placing the victim again in a power relationship with the helper.^29^, ^35^

During the help‐seeking process, women first usually turn to informal sources, specifically, family, friends or sometimes coworkers.^25^, ^29^, ^30^ The main reasons that lead them to seek help and try to leave the relationship are statements such as, ‘I couldn't stand it anymore’; the fear or actuality of death threats; concern about the negative effects on their children; or an escalation of violence.^29^, ^30^, ^36^ In contrast, the reasons they do not seek help, decide to stay or return to the relationship, or the difficulties they face leaving the relationship are difficulty identifying the violence (perceiving it as normal or not serious), insufficient financial resources, having children, lacking social support, external pressures from the abuser and/or the immediate environment, believing that they will be stigmatized, hoping that it will change, having adverse feelings towards themselves such as helplessness or guilt, or emotional ambivalence (being in love).^8^, ^42^

Help‐seeking is not a uniform or linear process. It is influenced by geographical location, culture, available resources, immigration status, previous experiences of abuse, and the responses received when help was sought, among others.^30^, ^36^, ^43^ Furthermore, in most cases, the woman is still experiencing violence while she tries to obtain help, whether formal or informal. Often, attempts to end the relationship are numerous and are followed by reconciliation.^30^, ^37^ Consequently, the responses these women receive from their environment shape the recovery process and can be a key factor in its success.^44^

## 5 SUPPORT WITH AN IMPACT ON RECOVERY

The process of leaving and recovering from a relationship marked by gender violence requires different types of support, as we previously highlighted. Such support may be formal or informal. Formal support includes specialized services such as the police, lawyers, crisis lines, shelters or health professionals.^3^, ^45^ Ansara and Hindin^30^ indicate that these types of resources are usually used when violence persists and is increasing. Informal support networks include family; friends; or people from the community, such as support groups. As we noted, a large number of studies agree that the most common sources of informal support for women who experience intimate partner violence are family and friends.^2^, ^30^, ^41^, ^42^

These formal or informal support networks work by helping women identify sources of support in their environment; helping them strengthen their ties with those supports and disconnect from toxic people; helping them identify their strengths and increase their awareness of the resources available to them; contributing to the improvement of their mental health; guiding them to rethink and reformulate their life history—especially regarding their affective‐sexual relationships—and providing practical support, such as help with accommodations, moving, childcare or financial aid.^2^, ^27^, ^28^ Many of these activities give the women time to think about their experience, an element that, as shown previously, is essential in recovery.

Regarding the impact of networks on interventions, informal social support networks are effective for reducing the risk of violence in low‐violence cases. However, Goodman, Dutton, Vankos and Weinfurt^43^ warn that this type of support is not productive in high‐violence situations. Roberts and Schenkman‐Roberts^44^ indicate that, in the case of the women who participated in their study, family intervention helped them end the relationship before the violence had taken root. Support groups contribute to physiological, psychological, economic or social improvement.^27^ For these positive effects to occur, useful responses by informal sources of support are as follows: encourage women to seek legal or emotional support/counselling, provide safe housing, or actively intervene (e.g. by calling the abuser to tell him to stop the abuse and to outline the possible repercussions for him if he does not).^2^

Regarding formal networks, Fugate, Landis, Riordan, Naureckas and Engel^8^ describe three important implications that service professionals should consider: the need to better communicate the services available, the need to assess said services, and the analysis of whether the services are suited to the diversity of profiles and situations that violence encompasses. To maximize the potential benefits of each service, coordination among them is necessary.^45^ This coordination should be aimed at collaboration, with a consideration of the work being done from different perspectives. Sinko, Burns, O'Halloran and Saint Arnault^22^ suggest professionals be trained in appropriate therapies and responses. They also propose that, to help women move forward, professionals should shift their focus from constructing negative responses to violence to making healing the survivor's primary goal.

In Choi and Ann's^12^ research on formal networks, they observed that 80% of professional interventions had been carried out in medical care settings. However, it is unclear whether this situation is the same in all countries or whether different factors, such as the social policies applied, could alter these results. In addition, interventions carried out by different disciplines may not use the same approach; therefore, we should explore the interventions developed by social workers or NGOs. In their research, Choi and Ann^12^ also identified the predominance of five types of intervention: (a) screening and assessment, (b) identification, (c) referral, (d) other behavioural outcomes and (e) multiple behavioural outcomes.

### 5.1 Informal social support

Informal support is vital given its potential positive impact on recovery and the fact that it is the first resort for women in cases of gender violence. Therefore, we must examine how people who witness or are aware of a situation of gender violence react and what motivates their support. We can classify the reactions into four types^46^, ^47^: active participants (those who join in the aggression), non‐responsive bystanders, those who intervene to defend the victim, and those who alert other people to intervene on behalf of the victim. To understand this typology, the work of Flecha^46^ highlights three elements that condition the reaction of those who witness or are aware of a situation of gender violence: their knowledge about the subject, their relationship with the victim or the aggressor, and the perception of what may happen to them after they offer their help. Regarding this last element and with reference to the work of Dziech and Weiner,^48^ Flecha incorporates a new typology into the conceptualization of gender violence: second order of sexual harassment (SOSH).^46^ SOSH is a type of violence triggered by attempts to help victims of gender violence; its purpose is to stop the support and hide the violence. Thus, the fear of possible retaliation for assisting a woman in a situation of gender violence—the fear of experiencing SOSH—can significantly affect the reactions of people in the environment and may even paralyse them. This fear can also affect the victims themselves, leading them to hide violence from people in their environment to protect them from retaliation.^25^ Therefore, if we do not try to protect them, it will be difficult for us to extend informal support to first‐order victims of GBV.^46^

## 6 CONTRIBUTIONS TO GUIDE INTERVENTIONS

Most policies to address GBV have focused on professional intervention—formal networks—as in the case of World Health Organization.^21^ This drive for professional responses reflects the desire that policies be based on scientific evidence.^12^ However, the mere fact that interventions are performed by professionals does not guarantee they are scientifically based or ensure their quality. Similarly, intervention by non‐professionals does not imply a lack of scientific basis.

The results presented in this article indicate that overcoming GBV requires an effort based on a community model in which all possible sources of support collaborate and coordinate, including formal networks (services and institutional resources), informal social networks and the women themselves.^26^, ^27^, ^49^ Collaboration between formal services and informal support networks, especially family and friends, is particularly important because women tend to seek support from informal networks first. Therefore, sensitization work must be carried out with the community to raise awareness about its obligation and responsibility when providing support.^45^, ^50^ For this awareness to have an impact on the community and to enhance its intervention, mechanisms must be activated that prevent SOSH.^46^, ^51^ In some cases, the lack of intervention is not due to a lack of awareness but to the fear of possible negative consequences, retaliation, etc; in short, fear of SOSH.

At the same time, we must provide the necessary training so the first people from whom women seek help can give adequate support, provide appropriate responses and share knowledge about the available resources.^29^

### 6.1 Characteristics of interventions by support networks that facilitate the process of overcoming GBV

Through a review of multiple studies, we found that the creation of a life detached from violence is not accomplished solely by the women who suffer from GBV; it requires help from professionals and people in the woman's environment. Part of the success in this process depends on the interactions established with these people.^21^ Our results highlight three aspects of these interactions that are of special relevance to the success of recovery.

#### 6.1.1 Do not blame the victim

All work aimed at overcoming GBV must begin with supporting the victims and rejecting violence, eliminating any type of blame of the victim herself. Making victims responsible for the violence they experience stigmatizes them in the eyes of the rest of society and reduces their chances of receiving support. Additionally, it can lead to greater isolation.^9^, ^10^, ^32^

In turn, reinforcing the image of the woman as guilty, or as a victim of some illness, is misleading and shifts the problem to the woman's personality, distracting the attention from why she is a victim of GBV and remains in the relationship.

#### 6.1.2 Value the transformative power of surviving women

Not blaming women for their situation should not lead us to think of them as being passive. Such an outlook occurs, for example, when the analysis focuses only on structural elements, which leads to the consideration that women cannot do anything about their affective relationships or everything surrounding the violence they suffer.^52^

Women who have experienced or are experiencing GBV should be treated as agents, and their capacity for transformation should be highlighted. For this to happen, their voices must be included in an egalitarian dialogue, reaching agreed‐upon interpretations of their reality and the goals of recovery, without their words being reinterpreted by experts.^12^, ^53^

#### 6.1.3 Promote reflection on socialization regarding affective‐sexual relationships

Despite the fact that a woman has ended a relationship, her socialization persists within the framework of the relationship of violence, which will affect her future choices. Therefore, encouraging reflection aimed at achieving a life free of violence requires women to review the narrative they have constructed around their affective‐sexual relationships. However, addressing gender stereotypes and submission is insufficient; this focus alone does not contribute enough knowledge to understand when a woman claims to be ‘in love’ with her abuser or to transform this situation.^53^ This reflection should go one step further by analysing attractiveness models linked to violence.^26^

When we understand that love and attraction are the result of socialization and therefore learning, the possibility of working on transformative resocialization emerges. This transformation involves working not only with women who have experienced GBV but also with the diversity of people that surround them and that can contribute to enhancing or rejecting attraction to violence models.^27^, ^28^, ^54^

## 7 CONCLUSIONS

One of the challenges in research on GBV is knowing how this recovery is achieved and how it can be maintained over time.^2^, ^3^ The previous scientific literature highlights two challenges for achieving GBV recovery: coping with the demands of life and managing post‐traumatic symptoms. Overcoming both will allow women to create a new life away from violence, having changed their views of themselves as victims and of relationships. If we take as a reference the classification established by Sinko and Saint Arnault,^21^ the three indicators that mark the achievement of these challenges are reconnection with oneself, with others, and with the world. Our analysis of the research leads us to conclude that this reconnection with others is key to progress in the other two indicators, since in no case is the leaving and recovery from GBV an individual process; it relies on a supportive environment.

In most cases, women experiencing GBV explain the situation to someone and seek help. Even so, a percentage of women (approximately 30%) do not seek help. Among the reasons are the perception of an adverse environment that blames them, does not support them, or is even complicit with those who exercise violence. In this regard, research is required to analyse in greater depth the reasons why these women do not seek help. In the case of women that explain the situation to someone, they usually turned to informal sources of support, such as family and friends.

The benefits of help range from finding the resources at their disposal to helping them reflect and build a new narrative about their life story, among others. For people around them to have an active role in supporting them, training the community to identify cases of violence or provide adequate responses will be insufficient; the SOSH must also be specifically combatted. Otherwise, some members of the potential support community may be discouraged from providing help.

Finally, we have identified three characteristics present in those interactions that positively impact the recovery processes of women in situations of GBV: not blaming them, highlighting the transformative potential of women, and reflecting on socialization in their sexual‐affective relationships.

These results as a whole are the basis for, in the context of research done through sol.net and subsequent research, delving into the characteristics of those interventions that have a greater impact on the recovery processes of women in GBV situations. The knowledge achieved so far is already very useful in enhancing the design and implementation of actions that can effectively contribute to facilitating the recovery of women, with the support of formal and informal networks.

## CONFLICT OF INTEREST

All authors declare they have no conflict of interest.
